# Supplementary material for: ChAdOx1 nCoV-19 (AZD1222) or nCoV-19-Beta (AZD2816) protect Syrian hamsters against Beta Delta and Omicron variants
Source: Nat Commun. 2022 Aug 8;13:4610. doi: 10.1038/s41467-022-32248-6 (PMC9358389; doi:10.1038/s41467-022-32248-6)
Supplement: Supplementary file 1 — Supplementary Information [file 41467_2022_32248_MOESM1_ESM.pdf]

SUPPLEMENTARY DATA

ChAdOx1 nCoV-19 (AZD1222) or ChAdOx1 nCoV-19-Beta (AZD2816) protect Syrian hamsters against the Beta, Delta, and Omicron variants of concern

Neeltje van Doremalen[1*], Jonathan E. Schulz[1], Danielle R. Adney[1#], Taylor A. Saturday[1], Robert J. Fischer[1], Claude Kwe Yinda[1], Nazia Thakur[2,3], Joseph Newman[2], Marta Ulaszewska[3], Sandra Belij-Rammerstorfer[3], Greg Saturday[4], Alexandra J. Spencer[3], Dalan Bailey[2], Colin A. Russell[5], Sarah C. Gilbert[3], Teresa Lambe[6], Vincent J. Munster[1*]

1. *Laboratory of Virology, National Institute of Allergy and Infectious Diseases, National Institutes of Health, Hamilton, MT, USA.*
2. *Viral Glycoproteins Group, The Pirbright Institute, Pirbright, Woking, UK*
3. *Pandemic Sciences Institute, Nuffield Department of Medicine, University of Oxford, Oxford, UK*
4. *Rocky Mountain Veterinary Branch, National Institute of Allergy and Infectious Diseases, National Institutes of Health, Hamilton, MT, USA*
5. *Laboratory of Applied Evolutionary Biology, Department of Medical Microbiology, Academic Medical Center, University of Amsterdam, Amsterdam, The Netherlands*
6. *Oxford Vaccine Group, Department of Paediatrics, University of Oxford, Oxford, UK and Chinese Academy of Medical Science (CAMS) Oxford Institute (COI), University of Oxford, Oxford, UK*

** = corresponding authors*

# Current affiliation = Lovelace Biomedical Research Institute, Department of Comparative Medicine, Albuquerque, NM, United States of America

Supplementary Table 1. Pathological scoring of lung tissue samples. The tissue slides were examined by a board-certified veterinary anatomic pathologist blinded to study group allocations. Scoring was done as follows. H&E; no lesions = 0; less than 1% = 0.5; minimal (1-10%) = 1; mild (11-25%) = 2; moderate (26-50%) = 3; marked (51-75%) = 4; severe (76-100%) = 5. IHC attachment; none = 0; less than 1% = 0.5; rare/few (1-10%) = 1; scattered (11-25%) = 2; moderate (26-50%) = 3; numerous (51-75%) = 4; diffuse (76-100%) = 5.

Beta VoC challenge							
		H&E			IHC		
Group	Day	% affected	Interstitial pneumonia	Bronchiolitis	% distribution	Type I and II pneumocytes	Bronchiolar epithelium
AZD2816	3	0	0	0	0	0	0
		0	0	0	0	0	0
		0	0	0	0	0	0
		0	0	0	0	0	0
		0	0	0	0	0	0
		0	0	0	0	0	0
	5	0	0	0	0	0	0
		0	0	0	0	0	0
		0.5	1	0	0	0	0
		0	0	0	0	0	0
		0	0	2	0	0	0
		0	0	0	0	0	0
AZD1222+ AZD2816	3	0	0	0	0	0	0
		0.5	0	2	0.5	0	3
		0	0	0	0	0	0
		0	0	0	0	0	0
		0.5	0	1	0.5	1	3
		0	0	0	0.5	0	2
	5	0	0	0	0	0	0
		0.5	1	0	0.5	1	2
		0.5	1	0	0	0	0
		0.5	1	0	0	0	0
		0.5	1	1	0.5	1	1
		0	0	0	0	0	0
Control	3	0.5	0	1	0.5	2	4
		0.5	1	1	0.5	2	4
		0.5	0	2	0.5	2	4
		0.5	0	1	0.5	2	4
		0	0	0	0.5	2	4
		0.5	0	2	0.5	2	4
	5	40	3	1	40	4	2
		40	3	2	40	4	3
		30	3	2	50	4	3
		40	3	2	30	4	3
		30	3	1	30	4	3
		30	3	1	30	4	4
Delta VoC challenge							
AZD2816	3	0.5	0	1	0.5	1	2
		0.5	0	1	0.5	0	3
		0.5	0	3	0.5	1	4
		0.5	0	2	0.5	1	3
		0.5	1	0	0.5	1	1
		0.5	0	2	0.5	0	2
	5	0	0	0	0	0	0
		0	0	0	0	0	0
		0	1	0	0	0	0
		0.5	0	0	0	0	0

		0	0	0	0	0	0
		0	0	0	0	0	0
AZD1222+ AZD2816	3	0	0	0	0.5	0	2
		0	0	1	0.5	1	4
		0	0	0	0.5	0	4
		0	0	0	0.5	1	2
		0.5	0	1	0.5	1	2
		0	0	0	0.5	0	1
	5	0	0	0	0	0	0
		0	0	0	0	0	0
		0	0	0	0	0	0
		10	2	0	0.5	1	0
		0	0	0	0	0	0
		0	0	0	0	0	0
Control	3	0.5	0	3	0.5	2	4
		0.5	0	3	0.5	2	4
		0.5	0	3	0.5	2	4
		0.5	0	3	0.5	2	4
		0.5	0	3	0.5	2	4
		0.5	0	3	0.5	2	4
	5	40	3	2	30	4	2
		40	3	2	30	4	2
		40	3	2	20	4	2
		50	3	2	30	4	2
		40	3	2	10	3	2
		40	3	2	30	4	2
Omicron VoC challenge							
AZD2816	3	0	0	0	0	0	0
		0	0	0	0	0	0
		0	0	0	0.5	0	1
		0	0	0	0	0	0
	5	0	0	0	0	0	0
		0	0	0	0	0	0
		0.5	1	0	0	0	0
		0	0	0	0	0	0
AZD1222	3	0	0	0	0.5	0	1
		0	0	0	0.5	0	1
		0	0	0	0.5	2	2
		0.5	0	1	0.5	0	1
	5	0	0	0	0	0	0
		0.5	1	0	0	0	0
		0	0	0	0	0	0
		0	0	0	0	0	0
Control	3	0	0	0	0.5	0	3
		0	0	0	0	0	0
		0.5	0	1	0.5	0	3
		0.5	0	1	0.5	0	3
	5	0	0	0	0.5	0	1
		0	0	0	0.5	0	1
		0.5	0	1	0.5	1	2
		0	0	0	0	0	0
Ancestral challenge							
AZD1222	3	0.5	0	1	0.5	1	3
		0	0	0	0.5	0	1
		0.5	0	2	0.5	0	2
		0.5	0	2	0.5	0	3
	5	0.5	1	0	0	0	0
		10	1	0	0.5	1	0
		0	0	0	0	0	0

		0.5	1	1	0	0	0
Control	3	0.5	1	3	10	3	4
		0.5	1	2	10	3	4
		0.5	1	3	10	3	4
		0.5	0	3	0.5	2	4
	5	50	3	2	60	4	1
		50	3	2	40	4	2
		40	3	2	40	4	3

33

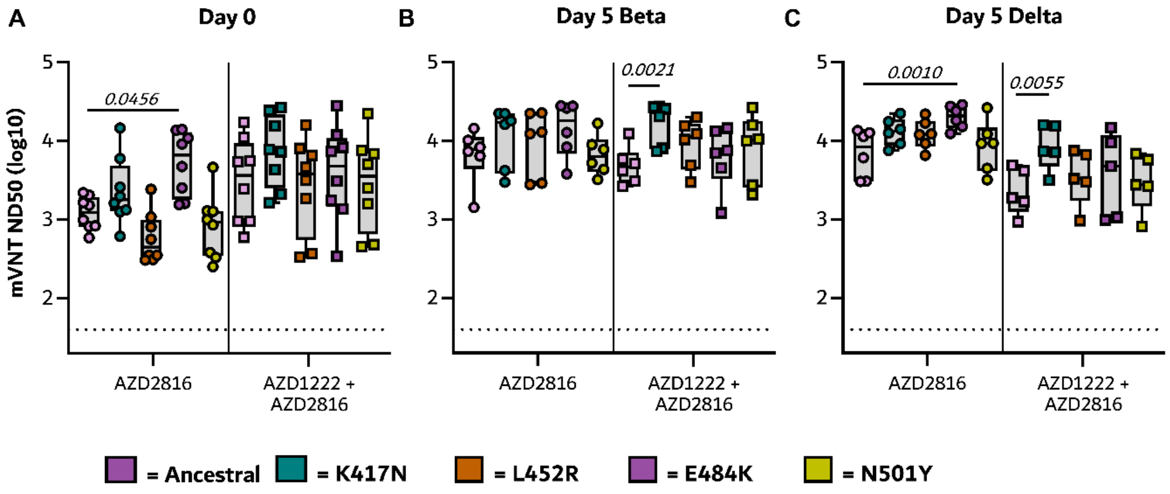

Supplementary Figure 1. Humoral response of vaccinated hamsters against single mutant pseudotypes. Boxplots (minimum to maximum) of binding antibody titers as measured by pseudovirus VN titers in hamster sera obtained on day 0 (left panel), day 5 after Beta VoC challenge (middle panel), and day 5 after Delta VoC challenge (right panel). Statistical significance was determined via a Friedman test followed by Dunn's multiple comparisons test comparing ancestral against mutant, p-values in italic when significant. N=6 per group, day 5 Delta prime boost group N=5. All boxplots are drawn from first quartile to third quartile, with a line at the median. Whiskers go from each quartile to minimum or maximum values. Source data are provided as a Source Data file.

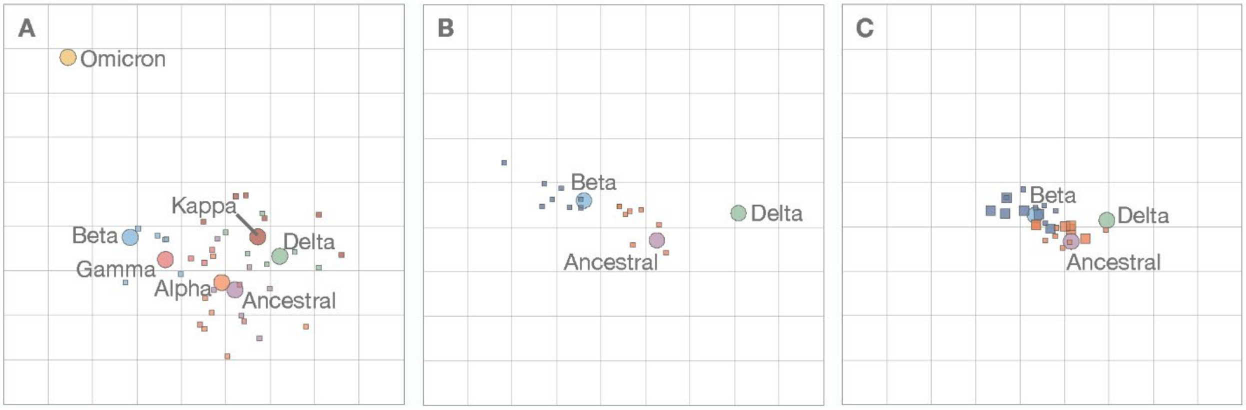

Supplementary Figure 2. Antigenic map using live VN assays. Multidimensional scaling was used to make maps showing the antigenic distance between different antigens and sera obtained from hamsters, based on live VN titers. (A) Mapping of live VN titers of sera obtained from hamsters challenged with ancestral virus, Alpha, Beta, Gamma, Kappa, Delta, or Omicron VoCs against the same VoCs. Antigens are shown as circles, sera is shown as squares. Sera is color-matched against antigen, e.g. sera obtained from hamsters challenged with the Beta VoC is blue. (B) Mapping of live VN titers of sera obtained from vaccinated hamsters at 0 days post challenge against ancestral virus, Beta, or Delta VoCs. Blue squares = AZD2816-vaccinated hamsters; Orange squares = AZD1222+AZD2816-vaccinated hamsters. (C) Mapping of live VN titers of sera obtained from vaccinated hamsters at 5 days post challenge against ancestral virus, Beta, or Delta VoCs. Blue squares = AZD2816-vaccinated hamsters; Orange squares = AZD1222+AZD2816-vaccinated hamsters; Large squares = hamsters challenged with Beta VoC; Small squares = hamsters challenged with Delta VoC. Source data are provided as a Source Data file.

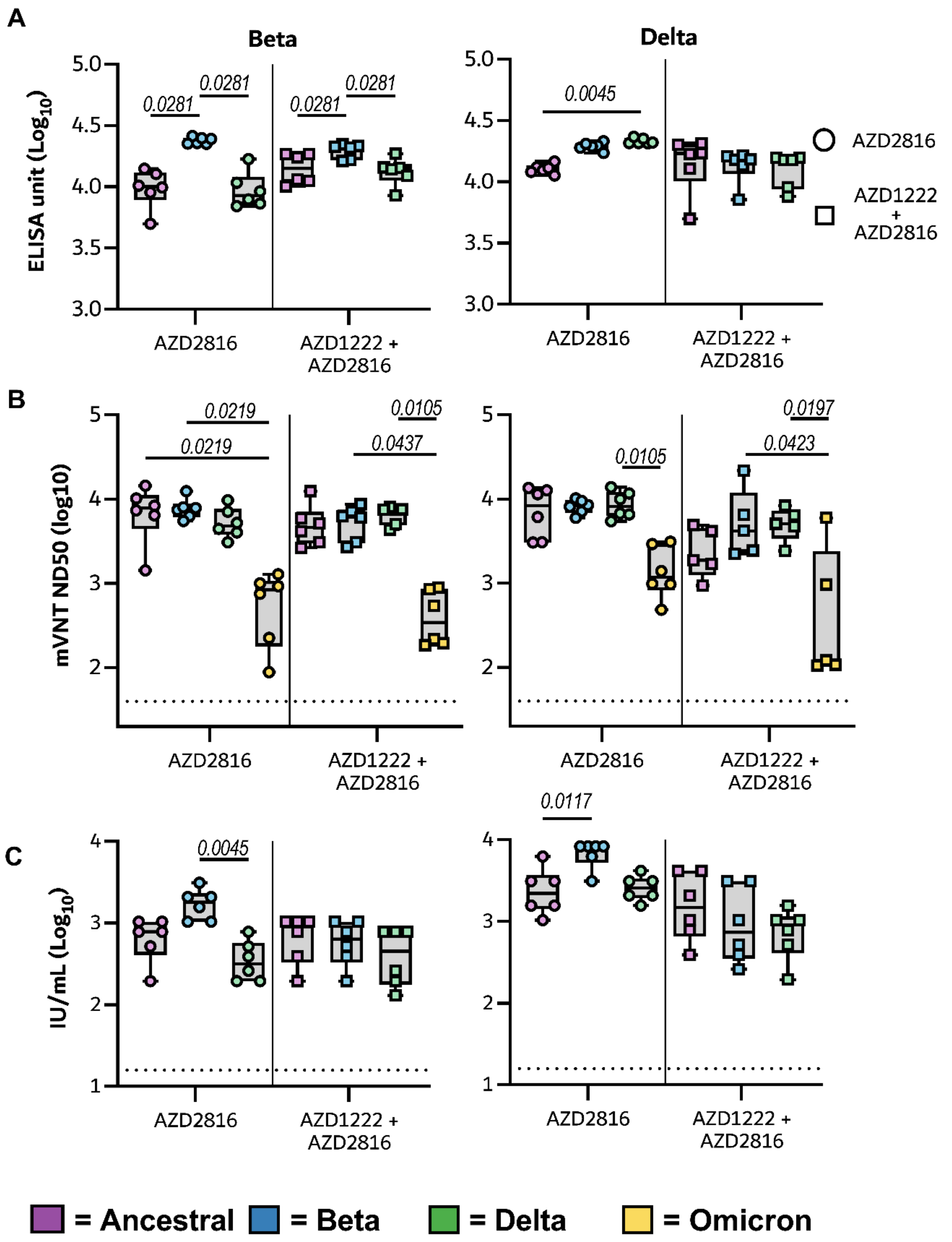

Supplementary Figure 3. Humoral response of vaccinated hamsters upon challenge with the Beta (left panels) or Delta (right panels) VoC. Boxplots (minimum to maximum) of binding antibody titers as measured by ELISA (A), pseudovirus VN titers (B), and live virus VN titers (C) in hamster sera obtained on day 5. Statistical significance was determined via a Friedman test followed by Dunn's multiple comparisons test, p-values in italic when significant. N=6 per group, N=5 for pseudovirus VN after Delta challenge. All boxplots are drawn from first quartile to third quartile, with a line at the median. Whiskers go from each quartile to minimum or maximum values. Source data are provided as a Source Data file.

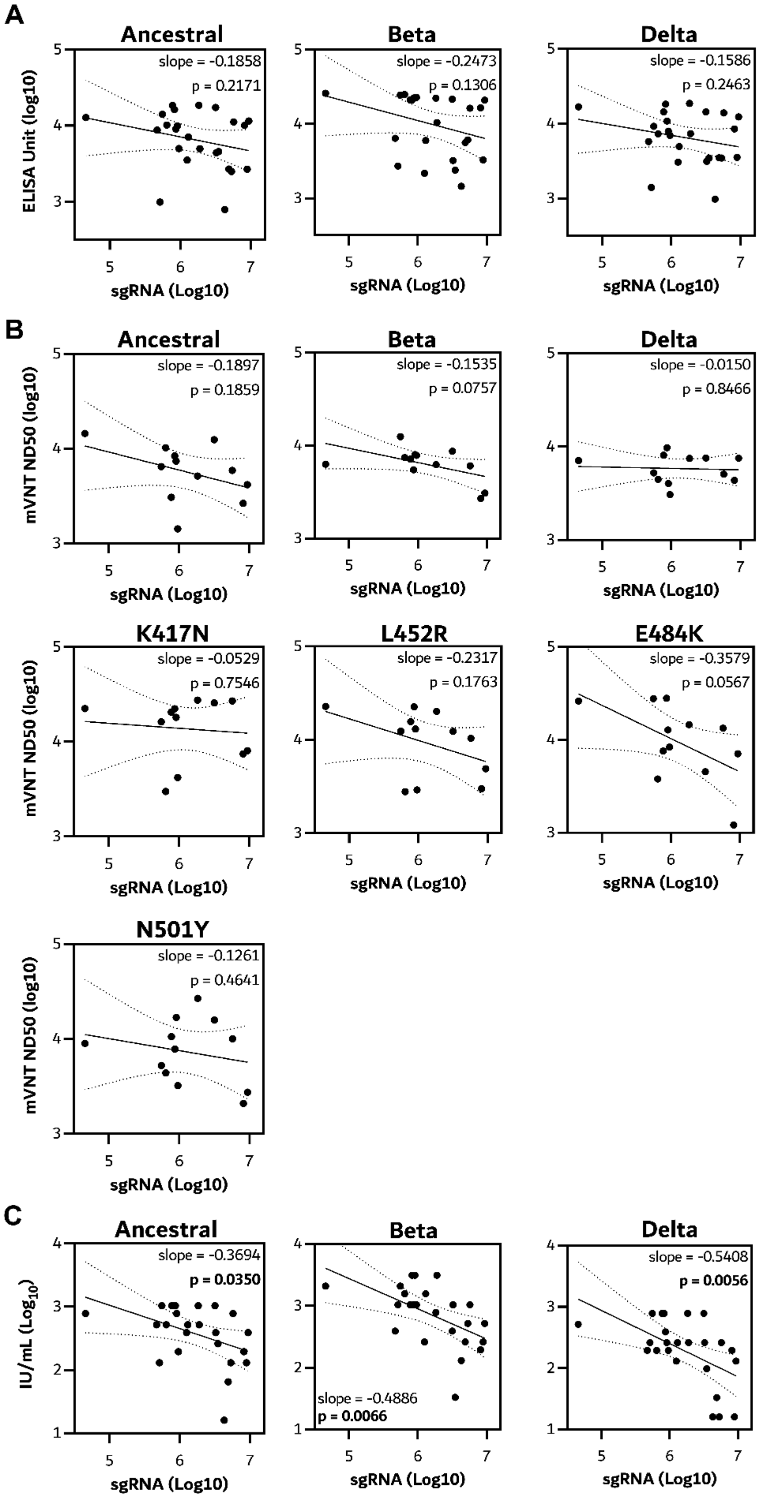

Supplementary Figure 4. Linear correlation plots between sgRNA load in oropharyngeal swabs and antibodies found in serum at day of necropsy post Beta challenge. A) Binding antibodies as determined via ELISA S. B) Pseudo VN titers. C) Live VN titers. Significance is calculated using simple linear regression. Source data are provided as a Source Data file.

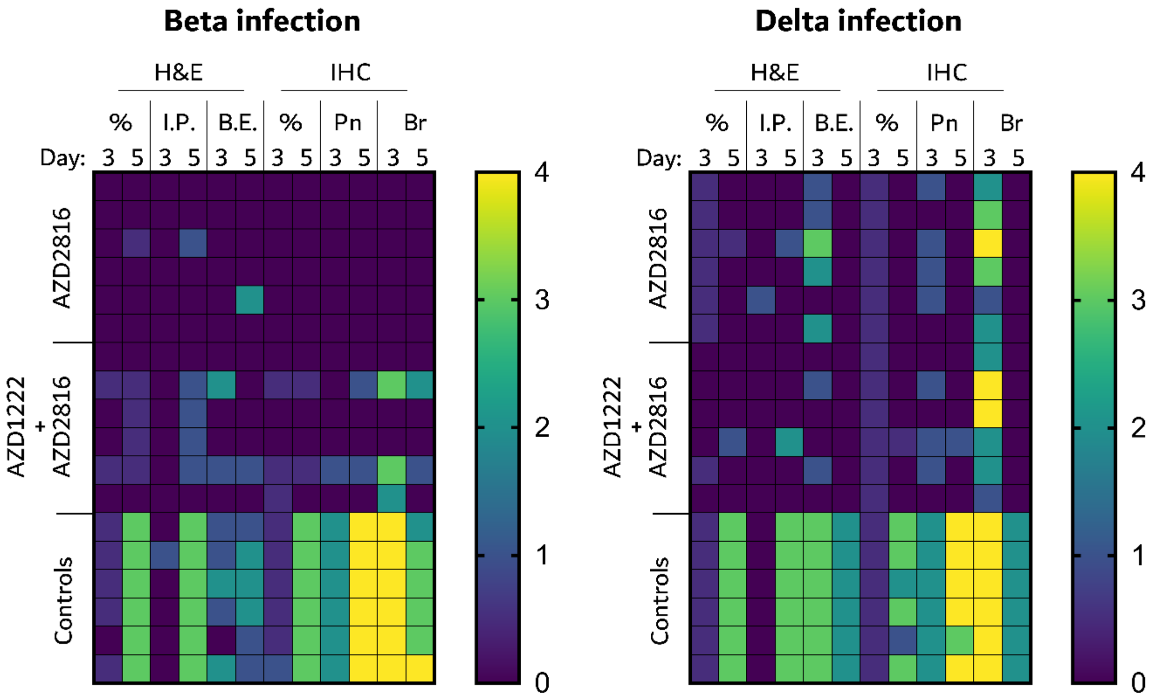

Supplementary Figure 5. Heatmap of scores of pathological features in lung tissue of hamsters infected with the Beta or Delta variant. Each square represents an individual score, each column represents a pathological feature at either day 3 or day 5. All features were scored 0 to 5. H&E = hematoxylin and eosin stain. IHC = Immunohistochemistry for SARS2 antigen. % = percentage affected. I.P. = interstitial pneumonia. B.E. = Bronchiolitis with epithelial cell necrosis. Pn = Staining of type I and type II pneumocytes. Br = Staining of bronchiolar epithelium. Source data are provided as a Source Data file.

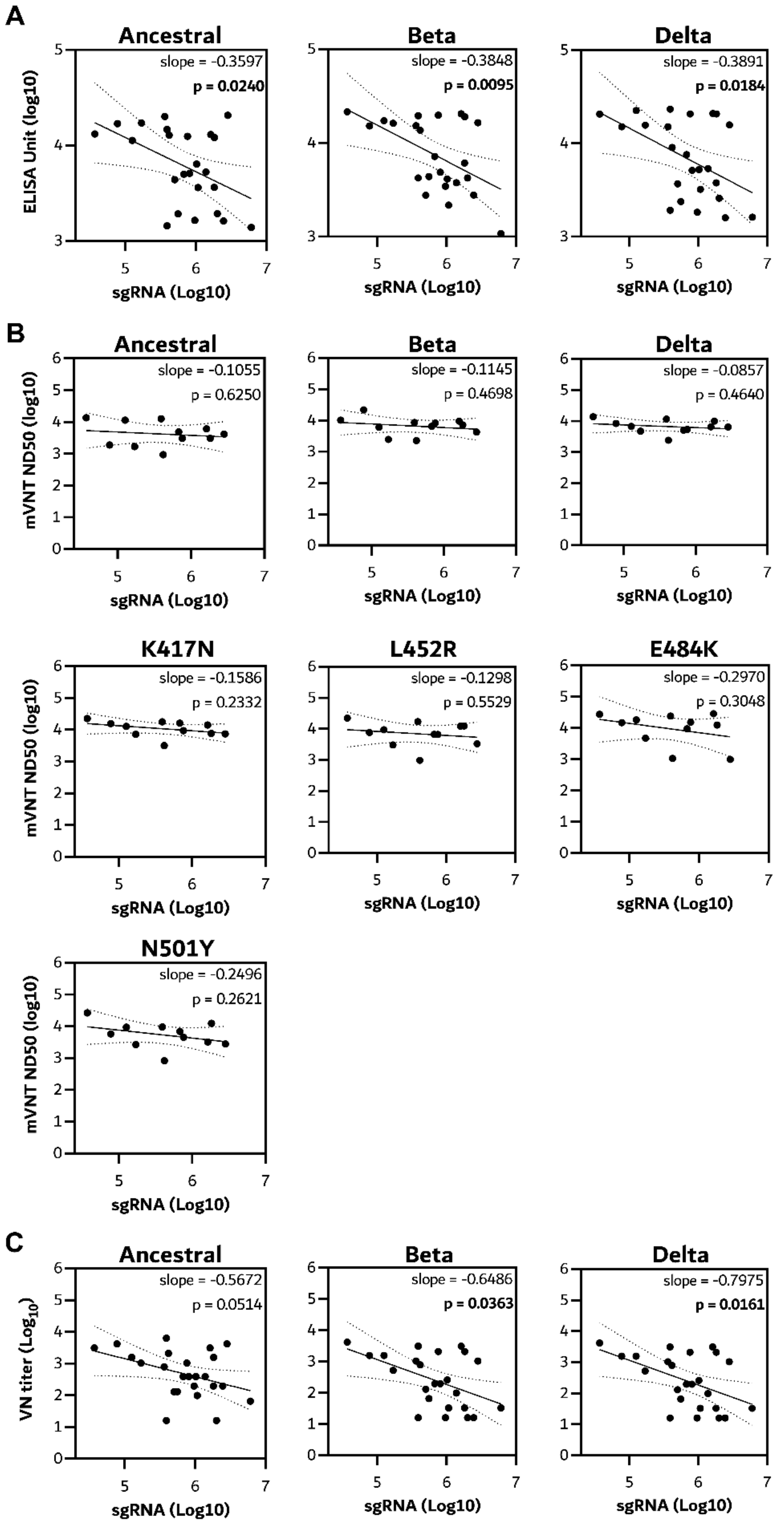

Supplementary Figure 6. Linear correlation plots between sgRNA load in oropharyngeal swabs and antibodies found in serum at day of necropsy post Delta challenge. A) Binding antibodies against Delta S. B) Pseudo VN titers against Delta. C) Live VN titers against the Delta variant. Significance is calculated using simple linear regression. Source data are provided as a Source Data file.

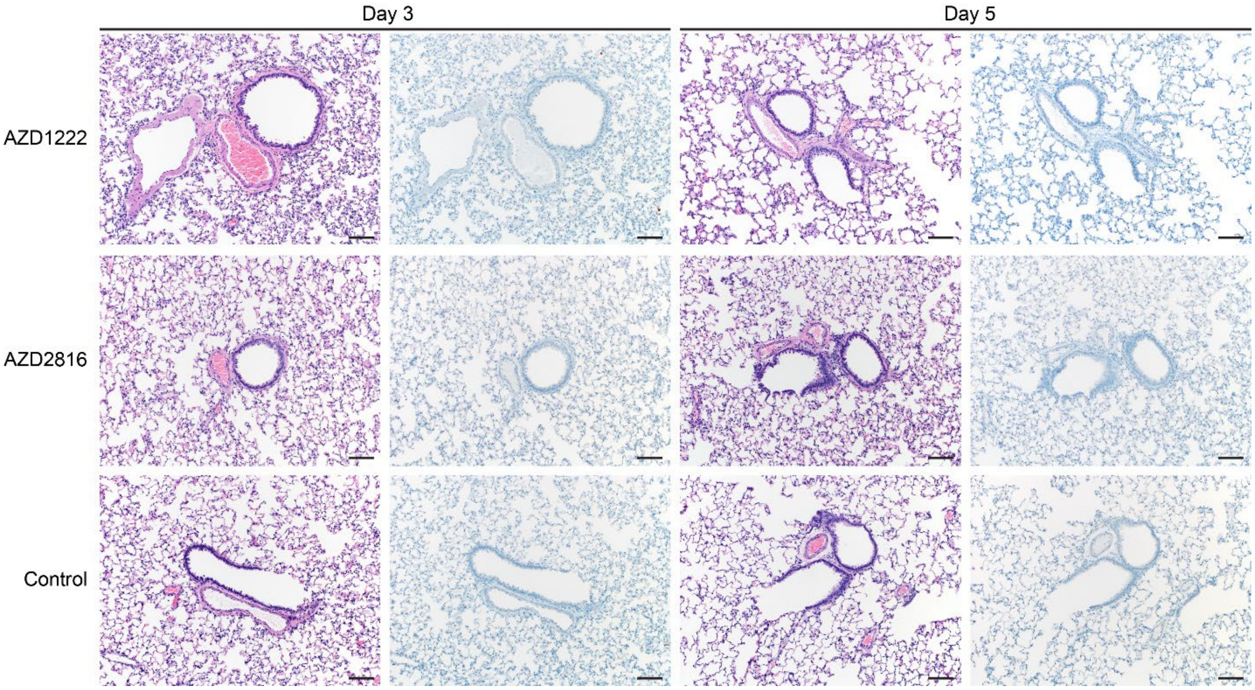

Supplementary Figure 7. Pulmonary effects of intranasal challenge with the Omicron VoC in vaccinated and control hamsters at day 3 and 5. H&E staining (1st and 3rd column) and IHC staining against N protein (brown, 2nd and 4th column), 100x, scale bar = 100 µm. N=4. Most vaccinated and control animals showed no pathology in the lower respiratory tract, except for minimal interstitial pneumonia on day 5 in 1/4 animals in each vaccine group. Antigen staining was limited to bronchial and bronchiolar epithelium in 2/4 control animals on day 5 and 1/4 AZD1222 vaccinated animals on day 3, as well as in type I and II pneumocytes in l/4 animals in control animals on day 5.
